# Cancer cell-soluble factors reprogram mesenchymal stromal cells to slow cycling, chemoresistant cells with a more stem-like state

**DOI:** 10.1186/s13287-017-0709-9

**Published:** 2017-11-07

**Authors:** Ahmed El-Badawy, Mohamed A. Ghoneim, Mahmoud M. Gabr, Radwa Ayman Salah, Ihab K. Mohamed, Marwa Amer, Nagwa El-Badri

**Affiliations:** 1 Center of Excellence for Stem Cells and Regenerative Medicine (CESC), Zewail City of Science and Technology, Sheikh Zayed District, 12588, 6th of October City, Giza, Egypt; 2 Urology and Nephrology Center, Mansoura University, Mansoura, Egypt; 3 Department of Zoology, Faculty of Science, Ain Shams University, Cairo, Egypt

## Abstract

**Background:**

Mesenchymal stem cells (MSCs) play different roles in modulating tumor progression, growth, and metastasis. MSCs are recruited to the tumor site in large numbers and subsequently have an important microenvironmental role in modulating tumor progression and drug sensitivity. However, the effect of the tumor microenvironment on MSC plasticity remains poorly understood. Herein, we report a paracrine effect of cancer cells, in which they secrete soluble factors that promote a more stem-like state in bone marrow mesenchymal stem cells (BM-MSCs).

**Methods:**

The effect of soluble factors secreted from MCF7, Hela, and HepG2 cancer cell lines on BM-MSCs was assessed using a Transwell indirect coculture system. After 5 days of coculture, BM-MSCs were characterized by flow cytometry for surface marker expression, by qPCR for gene expression profile, and by confocal immunofluorescence for marker expression. We then measured the sensitivity of cocultured BM-MSCs to chemotherapeutic agents, their cell cycle profile, and their response to DNA damage. The sphere formation, invasive properties, and in-vivo performance of BM-MSCs after coculture with cancer cells were also measured.

**Results:**

Indirect coculture of cancer cells and BM-MSCs, without direct cell contact, generated slow cycling, chemoresistant spheroid stem cells that highly expressed markers of pluripotency, cancer cells, and cancer stem cells (CSCs). They also displayed properties of a side population and enhanced sphere formation in culture. Accordingly, these cells were termed cancer-induced stem cells (CiSCs). CiSCs showed a more mesenchymal phenotype that was further augmented upon TGF-β stimulation and demonstrated a high expression of the β-catenin pathway and ALDH1A1.

**Conclusions:**

These findings demonstrate that MSCs, recruited to the tumor microenvironment in large numbers, may display cellular plasticity, acquire a more stem-like state, and acquire some properties of CSCs upon exposure to cancer cell-secreted factors. These acquired characteristics may contribute to tumor progression, survival, and metastasis. Our findings provide new insights into the interactions between MSCs and cancer cells, with the potential to identify novel molecular targets for cancer therapy.

**Electronic supplementary material:**

The online version of this article (doi:10.1186/s13287-017-0709-9) contains supplementary material, which is available to authorized users.

## Background

Cancer cells alone cannot drive tumor growth or progression. An assemblage of normal tissue and bone marrow-derived stromal cells are recruited to constitute tumorigenic microenvironments [1]. Cancer progression seems to be mediated by a subgroup of these cells, mesenchymal stem cells (MSCs). MSCs are multipotent cells capable of differentiating into numerous cell types, including adipocytes, osteoblasts, chondrocytes, fibroblasts, and perivascular and vascular structures [2]. In addition to their high regenerative capacities [3, 4], MSCs have also been reported to be among the cells recruited in large numbers to the stroma of developing tumors [5–8] and subsequently have important microenvironmental roles in modulating tumor progression and drug sensitivity [9–11]. Several studies have reported the effect of MSCs on cancer cells [10, 12], but the fate of MSCs in the tumor stroma and the effect of cancer cells on MSCs remain poorly understood. The long lifespan and self-renewal capacity of stem cells make them survive long enough to accumulate DNA damage to produce cancer cells [13]. In addition, committed progenitors can acquire self-renewal ability and function as CSCs [14, 15]. Although direct evidence for the initial cause of the transformation of adult stem cells into CSCs is lacking, extensive research shows that host–tumor interaction results in the production of proinflammatory cytokines and chemokines, believed to modulate the microenvironment to the benefit of tumor growth, invasion, and metastasis [16–20].

Herein, we report the results of assays designed to assess the effect of coculture of human cancer cell lines MCF7, Hela, and HepG2 with human bone marrow mesenchymal stem cells (BM-MSCs) on their functional properties, phenotypic characteristics, and gene expression profiles. Coculture of BM-MSCs with different cancer cell lines resulted in the generation of chemoresistant, sphere-like cells with many properties of pluripotent cells and CSCs. In this report, we refer to these cells as cancer-induced stem cells (CiSCs), generated directly from human BM-MSCs upon exposure to cancer cell lines.

## Methods

### Cells and coculture conditions

Human BM-MSCs and cancer cell lines MCF7, HepG2, and HeLa (ATCC, Manassas, VA, USA) were maintained in DMEM supplemented with 10% fetal bovine serum (FBS), streptomycin, and penicillin (Life Technologies, USA) at 37 °C in a humidified incubator containing 5% CO_2_. For coculture experiments, human BM-MSCs were seeded in the lower wells of a Transwell cell culture system (six-well type, high-density membrane with 0.4-μm pores; Greiner, Germany) and grown to 60–70% confluence for 24–72 hours. Cancer cell lines (MCF7, HeLa, and HepG2) (∼1 × 10^5^ cells) were each then seeded in the upper chambers (cell culture inserts) and cultured in DMEM supplemented with 10% FBS, streptomycin, and penicillin (Life Technologies, USA). After a 5-day incubation, the medium (DMEM supplemented with 10% FBS) was replaced with CSC medium as described previously [21, 22], consisting of DMEM/F12 medium (Life Technologies, USA), 2% B27 supplement (Life Technologies, USA), 20 ng/ml epidermal growth factor (EGF; Life Technologies, USA), 20 ng/ml basic fibroblast growth factor (bFGF; Life Technologies, USA), and 10 μg/ml insulin (Sigma-Aldrich, USA), and the upper chamber containing the cancer cells was removed.

### Flow cytometry characterization

For the flow cytometry analysis, cells were incubated in a blocking solution (PBS containing 1% BSA) for 10 minutes. After centrifugation, cells were resuspended in the blocking solution and stained with the following monoclonal antibodies for 30 minutes: FITC anti-CD44, PE anti-CD24, PerCP anti-CD19, APC anti-CD45, and FITC anti-CD34. For intracellular staining, cells were fixed in 4% paraformaldehyde, permeabilized with 0.1% Triton X-100, and blocked with 4% BSA. The cells were then stained with Oct-4 antibody (Cell Signaling Technology, USA), Sox2 antibody (R&D Systems, USA), Nanog antibody (Bioss Antibodies, USA), E-Cadherin antibody (Cell Signaling Technology, USA), N-Cadherin antibody (Abcam, USA), Snail + Slug antibody (Abcam, USA), ALDH1A1 antibody (Pierce Antibodies, USA), and β-Catenin antibody (Cell Signaling Technology, USA). Cells were then labeled with the appropriate Alexa Fluor® secondary antibodies (Molecular Probes, USA). Flow cytometry was carried out using FACSCalibur (Becton Dickinson, USA) following standard procedures with CellQuest Pro Software (Becton Dickinson, USA). Data analysis was performed using FlowJo v. 10.2 software (Treestar, USA) with super-enhanced Dmax (SED) subtraction analysis for determination of differences in histograms.

### Real-time qPCR

RNA was extracted using the PureLink® RNA Mini Kit (Life Technologies, USA) according to the manufacturer’s instructions and treated with DNAse I (Sigma-Aldrich). The cDNA was synthesized using the iScript™ cDNA Synthesis Kit (Bio-Rad, USA) and quantitative Real-Time PCR assay was performed using SsoAdvanced™ Universal SYBR® Green Supermix (Bio-Rad, USA) in the QuantStudio™ 12 K Flex Real-Time PCR System (Applied Biosystems, USA). The sequences of the used primers are indicated in Additional file 1: Table S1. The relative gene expression was calculated by the 2^–ΔΔCT^ method and the β-actin gene was used to normalize the data. Each reaction was performed in triplicate, and each experiment was performed twice.

### Confocal fluorescence microscopy immunostaining

To determine the changes in cytoskeleton structure and expression of different markers, CiSCs, BM-MSCs, and MCF7 cells were seeded on glass slides precoated with poly-d-lysine (Sigma-Aldrich, USA). Cells were fixed in 4% paraformaldehyde, permeabilized with 0.1% Triton X-100, and blocked with 4% BSA. Cells were then stained with Alexa Fluor® 488 Phalloidin (Molecular Probes, USA), α-tubulin antibody (Cell Signaling Technology, USA), Ki-67 antibody (Cell Signaling Technology, USA), Oct-4 antibody (Cell Signaling Technology, USA), Sox2 antibody (R&D Systems, USA), Nanog antibody (Bioss Antibodies, USA), E-Cadherin antibody (Cell Signaling Technology, USA), N-Cadherin antibody (Abcam, USA), Snail + Slug antibody (Abcam, USA), ALDH1A1 antibody (Pierce Antibodies, USA), and β-Catenin antibody (Cell Signaling Technology, USA). Cells were labeled with the appropriate Alexa Fluor® secondary antibodies (Molecular Probes, USA) and counterstained with Hoechst 33342 (Molecular Probes, USA) to visualize the cell nucleus. Cells were imaged either under a 60× or 100× objective with a Nikon A1R inverted laser scanning confocal microscope (Nikon microsystems, France).

### Chemotherapy sensitivity assay

CiSCs, BM-MSCs, MCF7 cells, and Hela cells were plated in a 12-well plate at a density of 4 × 10^5^ cells per well. Cells were then treated with cisplatin (at concentrations of 5, 10, 15, 20, and 25 μM) or doxorubicin (2, 6, and 10 nM). After incubation for 24 hours, the viability and apoptosis induced by anticancer regimens was analyzed by flow cytometry using an Annexin-V-FITC and propidium iodide (PI) apoptosis detection kit (Miltenyi Biotec Inc., USA) according to the manufacturer’s protocol. Experiments were performed three times in triplicate each.

### Cell cycle analysis

CiSCs, BM-MSCs, and MCF7 and Hela cells were collected in ice-cold PBS and fixed by chilled 70% ethanol overnight at 4 °C. These cells were then stained in PBS containing 100 μg/ml propidium iodide (Sigma Aldrich, USA) and 20 μg/ml RNase A (Thermo Fisher Scientific, USA). Flow cytometry was carried out using a FACSCalibur (Becton Dickinson, USA) following standard procedures and analyzed using CellQuest Pro Software (Becton Dickinson, USA).

### Single-cell gel electrophoresis assay (Comet assay)

DNA damage repair in response to different concentrations of chemotherapeutic agents was assessed by single-cell gel electrophoresis assay under alkaline conditions as described previously [23] with slight modifications. After treatment with cisplatin, CiSCs, BM-MSCs, and cancer cells were harvested and mixed with 1.3% low-melting agarose and the mix immediately placed onto frosted glass slides precoated with 0.6% agarose. After the agarose was solidified, slides were incubated in prechilled fresh lysis solution (2.5 M NaCl, 100 mM EDTA, 10 mM Tris-base, 1% Triton X-100, and 10% DMSO; pH 10.0) for 1 hour at 4 °C. Slides were placed in a reservoir filled with fresh prechilled alkaline electrophoresis buffer (300 mM NaOH, 1 mM ethylenediaminetetraacetic (EDTA) acid, pH > 13) for 30 minutes and then subjected to electrophoresis for another 30 minutes (25 V, 300 mA), followed by neutralization in 400 mM Tris–HCl (pH 7.5) for 30 minutes. Finally, DNA was stained with propidium iodide (2.5 μg/ml in PBS) for 30 minutes and imaged under a Leica DMi8 fluorescent microscope (Leica Microsystems, Germany). Average tail moments from 50 cells per sample were measured using Comet Assay IV software (Perceptive Instruments, UK).

### Sphere formation assay

The sphere formation assay was performed as described previously with slight modifications [24]. Single-cell suspensions of CiSCs were plated in ultralow-attachment flasks in DMEM-F12 2% B27 supplement (Life Technologies, USA), 20 ng/ml EGF (Life Technologies, USA), 20 ng/ml bFGF (Life Technologies, USA), 10 μg/ml insulin, and 10 μg/ml hydrocortisone. Spheres were cultured for 8 days, and then the cells collected from nonadherent cultures were quantified with a Bio-Rad TC20™ Automated Cell Counter (sizing range 20–336 μm). Experiments were performed three times in triplicate each.

### Invasion assay

Cell invasion assays were carried out in Transwell chambers with 8-μm pore polycarbonate filter inserts for six-well plates (Greiner, Germany). Inserts were coated with 1000 μl of ice-cold basement membrane matrix (Geltrex, Invitrogen) diluted 1:6 in DMEM/F12 and incubated for 1 hour at 37 °C. Cells (1.0 × 10^4^) were seeded in serum-free medium into the upper chamber and were allowed to invade toward the lower chamber with 10% FBS as the chemoattractant. After 24 hours of incubation at 37 °C, noninvasive cells were removed from the upper chamber with a cotton swab and the invading cells on the underside were fixed with 70% ethanol for 10 minutes and stained with 0.2% crystal violet for 15 minutes. Images were acquired using a Leica DMi8 phase-contrast microscope (Leica Microsystems, Germany) at 20× magnification. Ten independent fields were analyzed using ImageJ software and the experiments were done in triplicate.

### Surface ultrastructure characterization by electron microscopy

Briefly, cells were rapidly fixed in 0.1 M cacodylate buffered 2% glutaraldehyde for 2 hours at 4 °C, and then washed in equal volumes of sucrose 0.4% and cacodylate 0.2% for 2 hours before they were post-fixed in equal volumes of osmic acid 2% and cacodylate 0.3% for 1 hour. Afterward, the cells were washed twice with distilled water. Dehydration was carried out in an ascending series of ethyl alcohol for 5 minutes each (30%, 50%, 70%, and 90%) and then absolute alcohol 100% for 10 minutes three times and examined on a Formvar coating grid by environmental scanning electron microscope (Inspect S50; FEI, Holland).

### In-vivo xenotransplantation studies in nude mice

All animal procedures were carried out at the Urology and Nephrology Center Animal House in accordance with the institutional and National Institute of Health guidelines for the care and use of laboratory animals. The study protocol was approved by the ethical committee of Mansoura University. Nude mice (Swiss Nu/Nu; Charles River Laboratories, Paris, France) were housed as one mouse per cage. The mice (*n* = 5 per group) were anesthetized by intraperitoneal injection of ketamine (100 mg/kg) and diazepam (5 mg/kg). A total of 1 × 10^6^ CiSCs were implanted under the kidney capsule. After 2 months, the mice were euthanized and the kidneys were stained for histological analysis.

### Statistical analysis

All of the data are presented as the mean ± SD. An unpaired two-tailed Student *t* test was used to calculate the *P* values. *P* < 0.05 was considered statistically significant.

## Results

### Generation of CiSCs from adult BM-MSCs

**Fig. 1:**
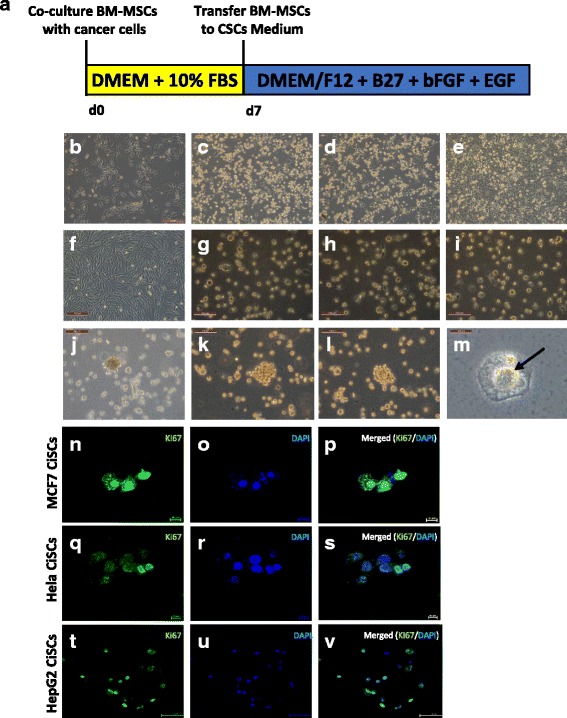
Generation of CiSCs from adult BM-MSCs. **a** Schematic illustrating the time schedule and derivation of CiSCs from BM-MSCs. **b** Morphology of BM-MSCs at day 0 cultured in standard conditions. **c**, d BM-MSCs after 2 days in coculture with (**c**) MCF7, (**d**) Hela, and (**e**) HepG2 cells. **f** Morphology of BM-MSCs at day 5 cultured in standard conditions without cancer coculture. **g**–**i** BM-MSCs after 5 days in coculture with (**g**) MCF7, (**h**) Hela, and (**i**) HepG2 cells showing generation of spheroid-like cells. **j**–**l** CiSCs growing in colonies in suspension. **m** Central cavity formation (arrow) becomes evident in CiSCs after several weeks in culture. **n**–**v** Confocal immunofluorescence images for Ki-67 of (**n**–**p**) MCF7, (**q**–**s**) Hela, and (**t**–**v**) HepG2 CiSCs. Nuclei stained with DAPI (blue). BM-MSC bone marrow mesenchymal stem cell, CiSC cancer-induced stem cell, CSC cancer stem cell, bFGF basic fibroblast growth factor, DMEM Dulbecco’s modified Eagle’s medium, EGF epidermal growth factor, FBS fetal bovine serum

The protocol for induction of human CiSCs from BM-MSCs is summarized in Fig. 1a. Human BM-MSCs were cocultured with different cancer cell lines using a Transwell culture system, which allowed for the exchange of soluble mediators yet segregated the cells. After 2 days of coculture, BM-MSCs began to form 3D colonies in suspension (Fig. 1c–e). Subsequently, outgrowth of cells in these 3D colonies detached from the colonies and formed spheres in suspension. After 5 days, most of the cocultured BM-MSCs formed spheres (Fig. 1g–i). By contrast, parental BM-MSCs did not form such 3D colonies or spheres (Fig. 1f). On day 5, the cells were transferred into low-attachment flasks and the medium (DMEM containing 10% FBS) was replaced with a CSC-specific culture medium (DMEM/F12 with 2% B27 supplement, 20 ng/ml EGF, 20 ng/ml bFGF, and 10 μg/ml insulin). When transferred to plates that do not permit adherence, these spheres could be maintained in suspension and grew in colonies (Fig. 1j–l). During this period, they increased in size and formed a central cavity (Fig. 1j–m). The proliferation capacity of the generated CiSCs was confirmed by positive immunostaining for Ki-67, a proliferation marker (Fig. 1n–v).

### CiSCs express human embryonic stem cell-specific markers

**Fig. 2:**
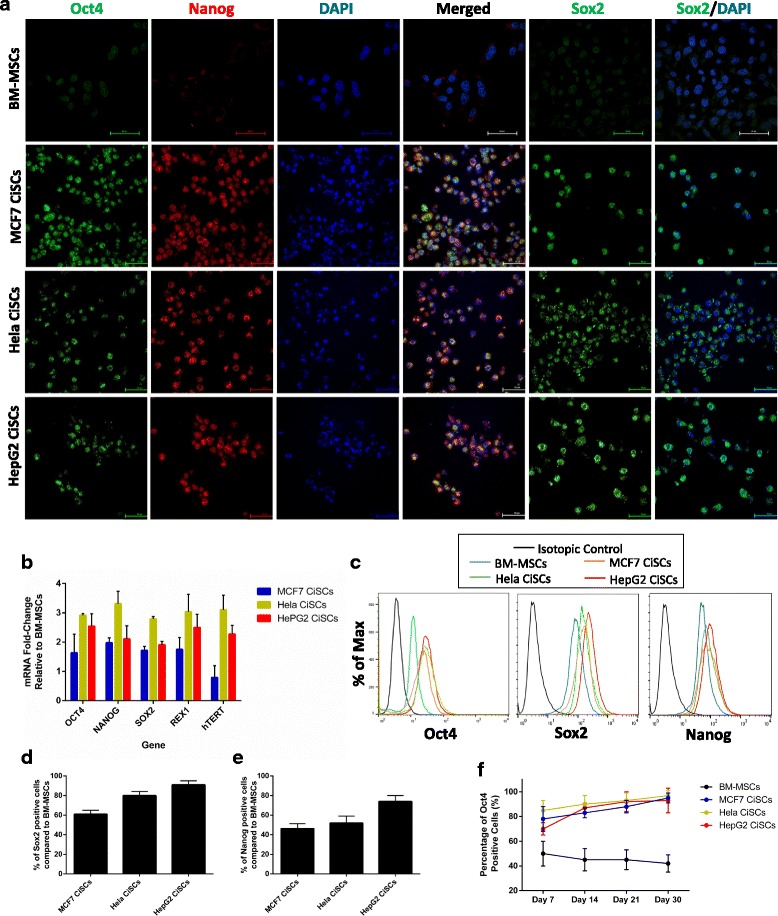
CiSCs express human ESC-specific markers. **a** Confocal immunofluorescence images for Oct4 (green), Nanog (red), and Sox2 (green) of control BM-MSCs and MCF7, Hela, and HepG2 CiSCs. Nuclei stained with DAPI (blue). Scale bars = 60 μM. **b** Expression levels of mRNAs encoding OCT4, NANOG, SOX2, REX1, and hTERT in MCF7, Hela, and HepG2 CiSCs relative to parental BM-MSCs determined by real-time qRT-PCR. Data reported on a log-10 scale as mean ± SD. **c** Flow cytometry overlay histogram analysis of Oct4, Sox2, and Nanog in BM-MSCs and MCF7, Hela, and HepG2 CiSCs. For comparison, isotype control (black) was used to define the positive and negative populations for each marker. **d** Oct-4 protein expression levels increase with the number of days (7–30) when cultured in the presence of B27, EGF, and bFGF, as determined by intracellular flow cytometry staining, indicating the self-renewal capacity of CiSCs. **e**, **f** Quantification of the percentage of (**e**) Sox2-positive and (**f**) Nanog-positive cells compared to parental BM-MSCs by intracellular flow cytometry staining. Proportions of positive cells measured by subtracting control parental BM-MSC staining from test histograms using super-enhanced Dmax (SED) normalized subtraction using FlowJo v. 10.2 software. Data presented as mean ± SD. BM-MSC bone marrow mesenchymal stem cell, CiSC cancer-induced stem cell

There is strong evidence that overexpression of embryonic stem cell (ESC) genes occurs in human cancers and is relevant to tumor formation, tumorigenicity, tumor metastasis [25], and tumor recurrence after chemotherapy or radiotherapy [26]. To investigate the pluripotency state of CiSCs, we used confocal immunofluorescence imaging to examine the expression of pluripotency markers, based on previously reported ESC markers [27]. CiSCs expressed higher levels of typical pluripotency markers including OCT4, NANOG, and SOX2 as shown by immunostaining (Fig. 2a). To investigate expression at the mRNA level, we performed real-time qPCR analysis and found a significant increase in mRNA expression levels of OCT4, SOX2, NANOG, and REX1 (Fig. 2b) in CiSCs compared to their parental BM-MSCs. Interestingly, telomerase reverse transcriptase (hTERT) increased up to 3-fold in CiSCs when compared to its levels in parental BM-MSCs (Fig. 2b).

To further confirm pluripotency marker protein expression levels, we performed intracellular flow cytometry staining. Our data showed increased expression of pluripotency markers (Fig. 2c–e). The level of Oct4 was further increased at 30 days in culture as shown by intracellular flow cytometry (Fig. 2f), suggesting that CiSCs could maintain their self-renewal capacities in vitro. These results suggest that at least some CiSCs are reexpressing pluripotency genes.

### Single-cell colony formation, sphere formation, and invasiveness of CiSCs

**Fig. 3:**
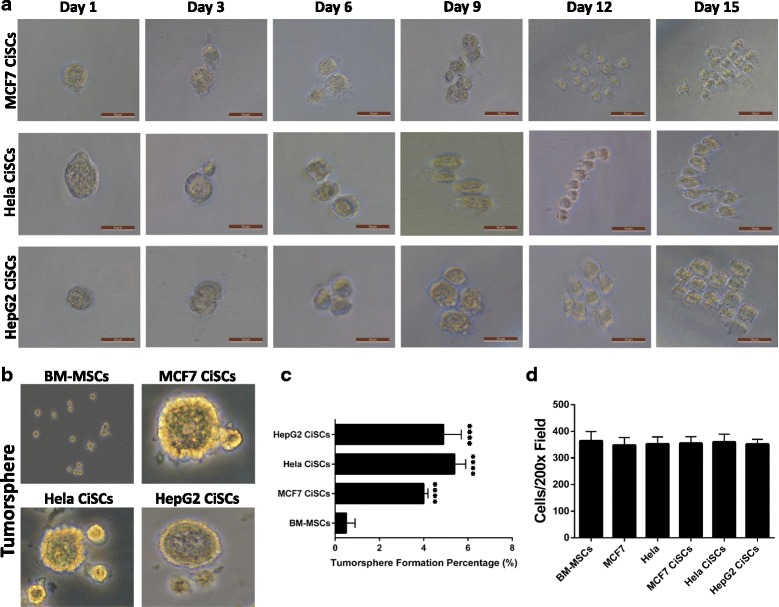
Single-cell colony formation, tumorsphere formation, and invasiveness of CiSCs. **a** Representative phase-contrast images of single CiSCs plated at a clonal density by limited dilution assays showing the colony-forming efficiency of a single CiSC. **b** Phase-contrast images of tumorspheres formed from BM-MSCs and MCF7, Hela, and HepG2 CiSCs. **c** Quantification of tumorsphere-forming ability of BM-MSCs and MCF7, Hela, and HepG2 CiSCs showing CiSCs to have significantly higher tumorsphere formation percentage (*P* < 0.05). Data presented as mean ± SD. **d** Quantification of invading cells toward lower chamber of the insert (average of 10 picture fields at 200× total magnification for each cell type). Data presented as mean ± SD. BM-MSC bone marrow mesenchymal stem cell, CiSC cancer-induced stem cell

A critical feature of stem cells is their capacity to self-renew and generate hierarchically organized structures in which their progeny loses their self-renewing capacity during differentiation [28, 29]. We thus assayed the capacity of a single CiSC to generate a large number of progeny by single-cell colony formation assay, as assayed to determine functional heterogeneity among cancer cells derived from lung, ovary, and brain tumors [30, 31]. We initiated a series of single-cell cloning experiments in 96-well plates, and each well contained a single cell as assessed by phase-contrast inverted microscopy. A single CiSC showed the capacity to form colonies and produce a large number of progeny, indicating their self-renewal and tumorigenic potential (Fig. 3a).

We examined the self-renewal capacity of CiSCs by sphere-forming ability in suspension culture, an in-vitro measure of stem cell activity [24]. CiSCs showed about a 10-fold increase in sphere-forming ability compared to the parental BM-MSCs (Fig. 3b, c). We then investigated the invasive capacity of the CiSCs, a critical factor involved in cellular metastasis [32, 33]. A Matrigel-coated, modified Boyden chamber was used to quantitatively evaluate cell invasion. As shown in Fig. 3d, CiSCs had a high invasive capacity, comparable to their parental BM-MSCs and MCF7 and Hela cancer cells.

### CiSCs express cancer and cancer stem cells markers and display properties of a side population

**Fig. 4:**
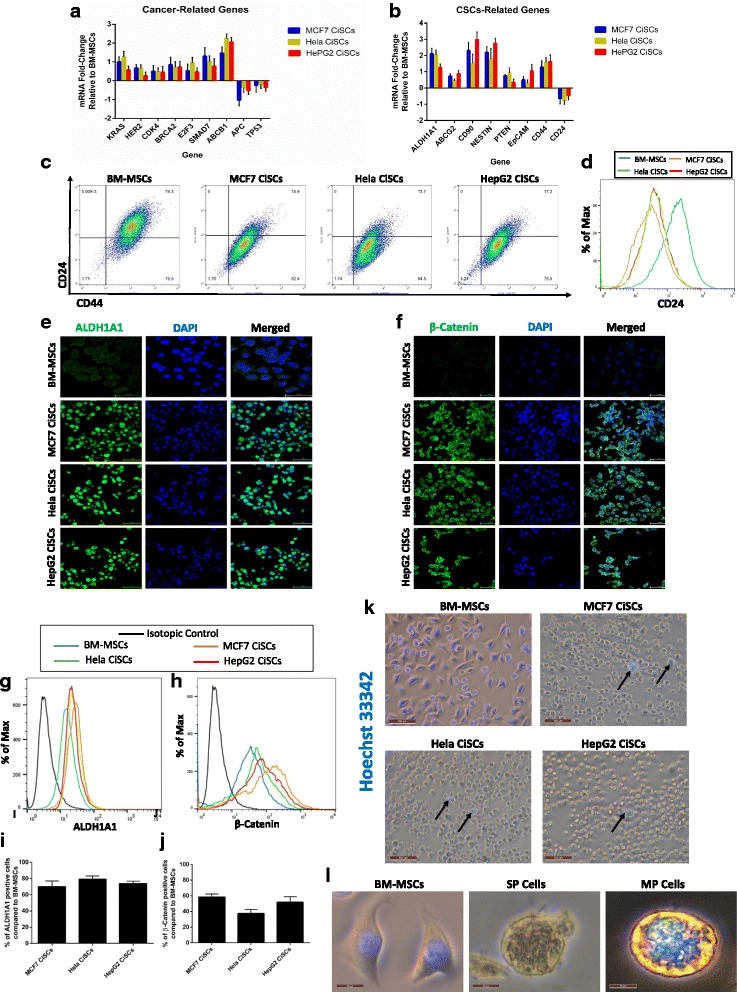
Expression of cancer and CSC markers in CiSCs and side population (SP) properties of CiSCs. **a** Expression levels of mRNAs encoding KRAS, HER2, CDK4, BRCA2, E2F3, SMAD7, ABCB1, APC, and TP53 in MCF7, Hela, and HepG2 CiSCs relative to the parental BM-MSCs determined by real-time qRT-PCR. **b** Real-time qRT-PCR analysis of CSC marker genes. β-actin mRNA used to normalize variability in template loading. Data reported on a log-10 scale as mean ± SD. **c** Flow cytometry plots for cell surface markers CD44 and CD24 in BM-MSCs and MCF7, Hela, and HepG2 CiSCs. Gating set to unstained control cells. **d** Flow cytometry overlay histogram analysis of CD24 expression in BM-MSCs and MCF7, Hela, and HepG2 CiSCs. **e** Confocal immunofluorescence images for ALDH1A1 (green) of control BM-MSCs and MCF7, Hela, and HepG2 CiSCs. Nuclei stained with DAPI (blue). Scale bars = 60 μM. **f** Confocal immunofluorescence images for β-catenin (green) of control BM-MSCs and MCF7, Hela, and HepG2 CiSCs. Nuclei stained with DAPI (blue). Scale bars = 60 μM. **g**, **h** Flow cytometry overlay histogram analysis of (**g**) ALDH1A1 and (**h**) β-catenin in BM-MSCs and MCF7, Hela, and HepG2 CiSCs. For comparison, isotype control (black) used to define the positive and negative population for each marker. **i**, **j** Quantification of percentage of (**i**) ALDH1A1-positive and (**j**) β-catenin-positive cells compared to parental BM-MSCs by intracellular flow cytometry staining. Proportions of positive cells measured by subtracting control parental BM-MSCs staining from test histograms using super-enhanced Dmax (SED) normalized subtraction using FlowJo v. 10.2 software. Data presented as mean ± SD. **k** Hoechst-positive staining of BM-MSCs and Hoechst-negative SP cells from MCF7, Hela, and HepG2 CiSCs. Arrows indicate small population of Hoechst-positive (MP) cells within the SP cells. **l** Higher power view of Hoechst-positive BM-MSCs, higher power view of a SP cell, and higher power view of Hoechst-positive (MP) CiSCs. BM-MSC bone marrow mesenchymal stem cell, CiSC cancer-induced stem cell, MP major population

To assess the “cancerous” status of the generated CiSCs, the expression of previously reported candidate cancer genes was compared to the parental BM-MSCs. qPCR results showed increased expression in mRNAs of KRAS, HER2, TP53, BRCA2, E2F3, APC, SMAD7, ABCB1, and CDK4 (Fig. 4a), which is associated with acquiring a cancerous phenotype. qPCR analysis showed increased expression of many cancer stem cell marker genes [34–36] such as ALDH1, ABCG2, CD90, NESTIN, PTEN, and EpCam. It is also of note that mRNAs of CD44 were increased and CD24 mRNA was downregulated (Fig. 4b). Flow cytometry analysis showed that more than 75% of cells converted from the CD44^+^CD24^+^ phenotype of the parental BM-MSCs into a CD44^+^CD24^low^ phenotype upon exposure to cancer cell-secreted factors (Fig. 4c, d), which is a pattern of expression seen in some cancer stem cells [37], suggesting that some of these cells may have acquired cancer stem cell properties.

Aldehyde dehydrogenase 1A1 (ALDH1A1) has been shown to be a potential marker of stemness, and also plays a role in the biology of CSCs [38, 39]. It has also been shown to play an important role in chemoresistance pathways, and its level was shown to correlate with the disease prognosis [40, 41]. Examination of the expression of ALDH1A1 in CiSCs at the protein level by immunofluorescence staining showed that CiSCs had a higher expression for ALDH1A1 in comparison with the parental BM-MSCs (Fig. 4e), and this increased expression of ALDH1A1 was confirmed by intracellular flow cytometry analysis (Fig. 4g, i).

To determine the possible molecular pathway(s) enabling the observed in-vitro change of BM-MSCs into the CiSC phenotype, we analyzed Wnt/β-catenin signaling in both cells. The Wnt/β-catenin signaling pathway is essential in the functioning of CSCs [42–45]. For instance, mammary stem cells with high levels of Wnt/β-catenin signaling have a much greater tumorigenic potential than their counterparts with low levels of Wnt/β-catenin signaling [46]. Moreover, Wnt/β-catenin signaling regulates CSC self-renewal, tumorigenesis, and cancer chemoresistance [47]. Our data showed increased cytoplasmic β-catenin expression in CiSCs in comparison to their parental BM-MSCs as shown by immunofluorescence confocal imaging, in which cytoplasmic β-catenin has been associated previously with poor outcome in breast cancer patients [48] (Fig. 4f). Using intracellular flow cytometry, β-catenin expression increased by approximately 50% compared to the parental BM-MSCs (Fig. 4h, j). These results suggest that Wnt/β-catenin signaling may be important in the conversion from BM-MSCs to CiSCs. These data show that soluble factors produced by cancer cells contribute to converting normal human BM-MSCs into cells with cancer stem cell characteristics.

CSCs are characterized by their ability to exclude Hoechst 33342 dye (and chemotherapy drugs) as they express multidrug-resistant transporters such as ABCG2, known as side population (SP) cells [49, 50]. We compared the CiSCs to their parental BM-MSCs for Hoechst dye exclusion. While parental BM-MSCs did not exclude the dye, more than 95% of sphere-derived CiSCs were Hoechst-negative (Fig. 4k, l), indicating their SP characteristics. Notably, these Hoechst-negative cells were much smaller (approximately 10 μm in diameter) than the major population (MP) cells, which consisted of Hoechst-positive cells (>20 μm). Taken together, it appears that CiSCs display properties of SP cells.

### CiSCs display resistance to chemotherapy and are slow-cycling

**Fig. 5:**
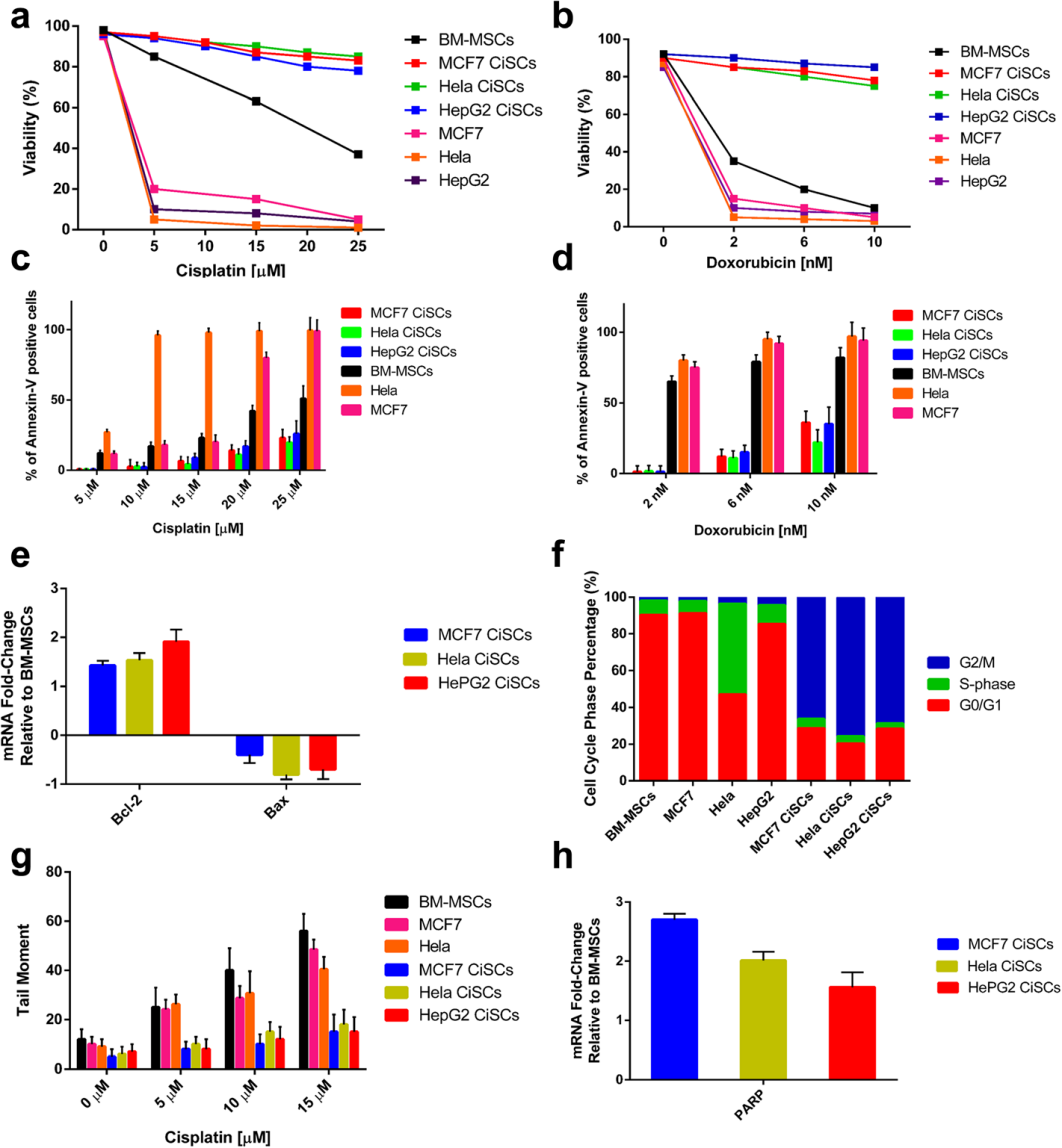
Drug sensitivity, DNA repair, and cycling profile of CiSCs. **a**, **b** CiSCs, parental BM-MSCs, and MCF7 and Hela control cells were exposed to increasing concentrations of cisplatin (**a**) or doxorubicin (**b**) for 24 hours. Cell viability determined by Annexin-V-FITC and PI apoptosis detection kit. CiSCs showed highly more significant resistant to cisplatin and doxorubicin than parental BM-MSCs (*P* < 0.05). **c**, **d** Percentage of Annexin-V-positive cells in MCF7, Hela, and HepG2 CiSCs, parental BM-MSCs, and MCF7 and Hela control cells upon exposure to increasing concentrations of cisplatin (**c**) or doxorubicin (**d**) for 24 hours, indicating that CiSCs exhibit significantly decreased apoptosis compared to parental BM-MSCs and control cancer cells (*P* < 0.05). Data presented as mean ± SD. **e** Real-time qRT-PCR analysis of Bcl-2 (anti-apoptotic protein) and Bax (pro-apoptotic molecule), indicating significantly increased expression of Bcl-2 and reduced expression of Bax in MCF7, Hela, and HepG2 CiSCs compared to parental BM-MSCs (*P* < 0.05). β-actin mRNA used to normalize variability in template loading. Data reported on a log-10 scale as mean ± SD. **f** CiSCs, parental BM-MSCs, and MCF7 and Hela control cells analyzed for their cell cycle profile and % population in G1/G0, S, and G2/M phases presented graphically. CiSCs appeared to show cell cycle arrest in the G2/M phase and have a slower cell cycle progress than their parental BM-MSCs. **g** CiSCs, parental BM-MSCs, and MCF7 and Hela control cells treated with increasing concentrations of cisplatin and presence of DNA damage assessed by single-cell gel electrophoresis assay under alkaline conditions (alkaline Comet assay). The average tail moment quantified showed CiSCs display significantly less DNA damage response upon exposure to increasing concentrations of cisplatin (*P* < 0.05). Data presented as mean ± SD. **h** Expression level of mRNA encoding poly-ADP-ribose polymerase (PARP), an essential protein involved in DNA repair, relative to parental BM-MSCs as determined by real-time qRT-PCR. Data reported on a log-10 scale as mean ± SD. BM-MSC bone marrow mesenchymal stem cell, CiSC cancer-induced stem cell

CSCs have been reported to be relatively resistant to chemotherapy [51]. Since CiSCs were shown to express markers of stemness and displayed SP cell properties, we investigated the response of the CiSCs and their parental cells to conventional chemotherapeutic agents using an Annexin-V-FITC and PI apoptosis detection kit. CiSCs were exposed for 24 hours to varying concentrations of cisplatin (0, 5, 10, 15, 20, and 25 μM) and for 24 hours to varying concentrations of doxorubicin (2, 6, and 10 nM) anti-cancer chemotherapeutic medications. Chemotherapy-induced cell death was significantly reduced in CiSCs relative to the parental BM-MSCs. CiSCs were more resistant than the parental BM-MSCs to two commonly used chemotherapeutic drugs, cisplatin (~40% increase) (Fig. 5a) and doxorubicin (~50% increase) (Fig. 5b). In response to cisplatin and doxorubicin chemotherapeutic agents, CiSCs displayed significantly lower Annexin-V positivity as compared to the parental BM-MSCs and the control MCF7 and Hela cells, indicating that CiSCs are more resistant to apoptosis (Fig. 5c, d). To investigate the possible mechanism enabling the CiSCs to block chemotherapy-induced apoptosis, we used qPCR to analyze the expression of Bcl-2 (an anti-apoptotic protein) and Bax (a pro-apoptotic molecule). Bcl-2 was overexpressed, while the pro-apoptotic molecule Bax was downregulated (Fig. 5e), suggesting that CiSCs block chemotherapy-induced apoptosis by preferential activation of the Bcl-2 cell survival response.

Several studies showed that the quiescent and slow-cycling stem cell population can frequently evade drug or radiation therapy rather than actively dividing cancer cells [51–53]. We thus analyzed the cell cycle status of the CiSCs and the parental BM-MSCs, as well as different cancer cell lines (MCF7 and Hela), and found remarkable difference in G0/G1 and G2/M phase cells (Fig. 5f). The CiSCs appeared to show slower cell cycle progress than their parental BM-MSCs.

One possible mechanism for the resistance of CSCs to chemotherapeutic agents is that the cells display a highly efficient DNA damage response, believed to contribute to their resistance to DNA-damaging chemotherapeutic agents [54–56]. We therefore sought to directly evaluate and quantify DNA damage in CiSCs in response to cisplatin using alkaline Comet assays. The parental BM-MSCs and MCF7 and Hela cancer cell lines were assayed in parallel as a positive control. Analysis of tail moments showed significantly elevated levels of DNA damage in the parental BM-MSCs and MCF7 and Hela cells compared to the CiSCs (Fig. 5 g). Compared to their parental BM-MSCs, qPCR analysis showed that CiSCs overexpress poly-ADP-ribose polymerase (PARP), which has an essential role in DNA repair (Fig. 5 h). Taken together, these data show that the slow-cycling CiSCs are relatively resistance to chemotherapeutic agents and this resistance maybe due to the ability of the CiSCs to protect the genome integrity by prompt activation of the DNA damage sensor and repair machinery.

### Generation of CiSCs from BM-MSCs is associated with an increase in the mesenchymal phenotype and microenvironment stimulation by TGF-β

**Fig. 6:**
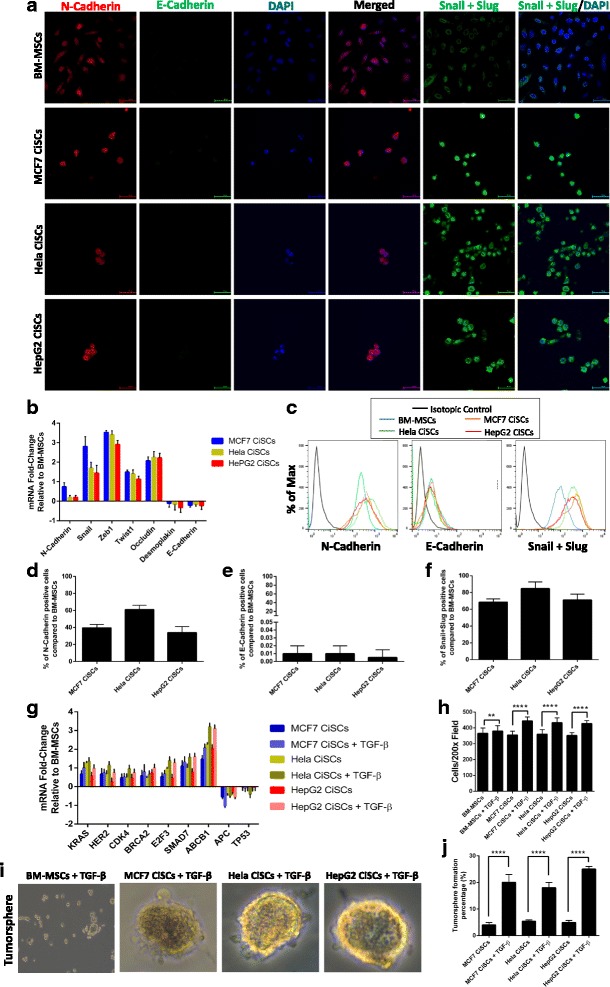
CiSCs exhibit a more mesenchymal phenotype compared to their parental BM-MSCs. **a** Confocal immunofluorescence images for N-Cadherin (red), E-Cadherin (green), and Snail + Slug (green) of MCF7, Hela, and HepG2 CiSCs. Nuclei stained with DAPI (blue). Scale bars = 60 μM. **b** Expression levels of mRNAs encoding N-Cadherin, Snail, Zeb1, Twist, Occludin, Desmoplakin, and E-Cadherin in MCF7, Hela, and HepG2 CiSCs relative to parental BM-MSCs as determined by real-time qRT-PCR. Data reported on a log-10 scale as mean ± SD. **c** Flow cytometry overlay histogram analysis of N-Cadherin, E-Cadherin, and Snail + Slug in BMMSCs and in MCF7, Hela, and HepG2 CiSCs. For comparison, isotype control (black) was used to define the positive and negative population for each marker. **d**–**f** Quantification of percentage of (**d**) N-Cadherin (**e**) E-Cadherin, and (**f**) Snail + Slug positive cells compared to the parental BM-MSCs by intracellular flow cytometry staining. Proportions of positive cells measured by subtracting the control parental BM-MSC staining from test histograms using super-enhanced Dmax (SED) normalized subtraction using FlowJo v. 10.2 software. Data presented as mean ± SD. **g** Expression levels of mRNAs encoding KRAS, HER2, CDK4, BRCA2, E2F3, SMAD7, ABCB1, APC, and TP53 in MCF7, Hela, and HepG2 CiSCs after treatment with 20 ng/ml of TGF-β for 7 days as determined by real-time qRT-PCR. Data reported on a log-10 scale as mean ± SD. **h** Quantification of invading CiSCs toward the lower chamber of the insert after treatment with 20 ng/ml of TGF-β for 7 days showing CiSCs to have a significantly higher invasive properties after treatment with TGF-β (**P < 0.05 and ****P < 0.001) (average of 10 picture fields at 200× total magnification for each cell type). **i** Phase-contrast images of tumorspheres formed from MCF7, Hela, and HepG2 CiSCs after treatment with 20 ng/ml of TGF-β for 7 days. **j** Quantification of tumorsphere-forming ability of MCF7, Hela, and HepG2 CiSCs after treatment with 20 ng/ml of TGF-β for 7 days showing CiSCs to have a significantly higher tumorsphere formation percentages after treatment with TGF-β (*P* < 0.05). Data presented as mean ± SD. BM-MSC bone marrow mesenchymal stem cell, CiSC cancer-induced stem cell, TGF-β transforming growth factor beta

Epithelial–mesenchymal transition (EMT), a process that has been associated with tumor recurrence, metastasis, and drug resistance [57, 58], has been recently tightly linked to the function and generation of CSCs [59–62]. Many studies have reported that cells undergoing EMT can acquire a stem cell-like state and showed an effective tumor-initiating ability, similar to CSCs [63–66]. Given the importance of EMT in acquiring a CSC phenotype, we investigated EMT markers in CiSCs to their parental BM-MSCs. Confocal immunofluorescence analysis of the expression of various transcription factors (EMT-TFs) known to control the EMT process showed that the expression of epithelial markers (such as E-Cadherin) was downregulated, while the expression of mesenchymal markers (such as N-Cadherin, vimentin, and fibronectin) was upregulated (Fig. 6a), as shown by confocal immunofluorescence imaging. When further characterized by real-time qPCR, CiSCs were highly positive for the mRNAs encoding mesenchymal markers (N-Cadherin and Snail + Slug) and were negative for the epithelial marker E-Cadherin (Fig. 6b). Furthermore, flow cytometry analysis showed that, relative to the expression levels in the parental BM-MSCs, the levels of N-Cadherin expression were increased, E-cadherin decreased, and the levels of Snail + Slug were increased strongly (Fig. 6c–f).

Extensive evidence indicates that activation of the EMT program and entrance into a stem cell state are generally triggered by contextual signals received by normal and neoplastic cells [67–69]. Among these signals, TGF-β has been shown to be a potent activator of the EMT program [70–72]. Accordingly, we examined whether TGF-β could further increase the expression of the cancerous markers reported previously in Fig. 2a. mRNAs of cancerous markers (KRAS, HER2, TP53, BRCA2, E2F3, APC, SMAD7, ABCB1, and CDK4) were all increased in CISCs stimulated with TGF-β (Fig. 6g). Furthermore, our data showed that TGF-β significantly increased the invasiveness of the CiSCs, compared with untreated CiSCs (Fig. 6h). Next, we functionally tested the effect of TGF-β on the stem cell activity of the CiSCs by tumorsphere-forming ability in 3D culture. After treating the CiSCs with 20 ng/ml of TGF-β for 7 days, the tumorsphere formation was increased by 5-fold in comparison with nonstimulated CiSCs (Fig. 6i, j).

### Multilineage differentiation of human CiSCs

**Fig. 7:**
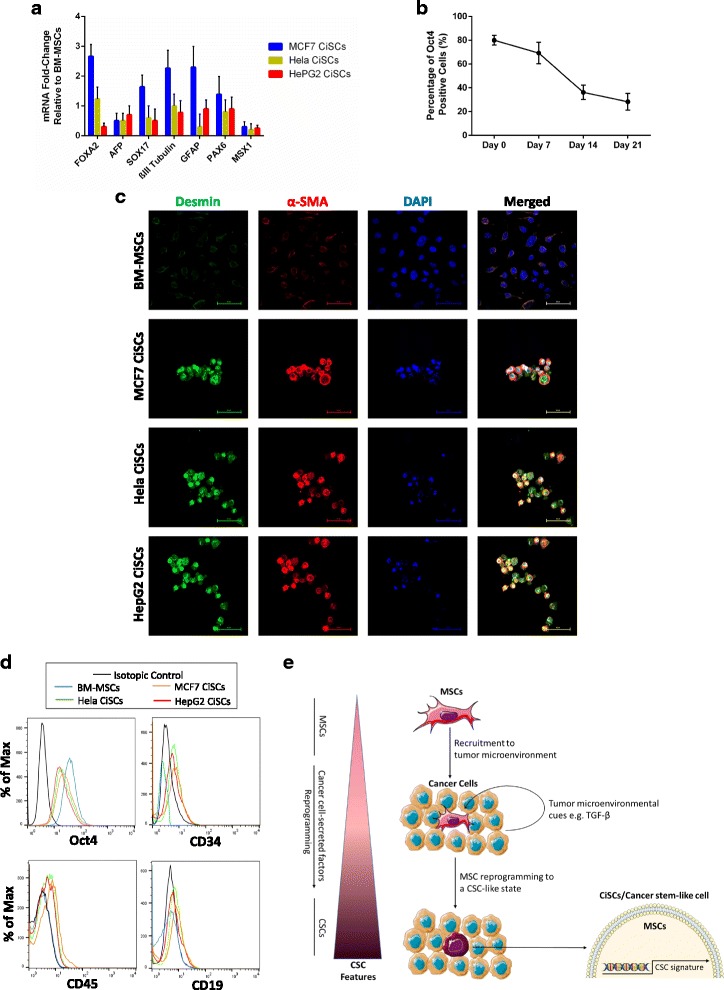
Differentiation of CiSCs reveals multilineage differentiation potential. **a** Real-time qRT-PCR analysis of various differentiation markers for the three germ layers indicating the multilineage differentiation potential of CiSCs. β-actin mRNA used to normalize the variability in template loading. Data reported on a log-10 scale as mean ± SD. **b** Oct-4 protein expression levels decrease with number of days (0–21) in culture when exposed to 10% FBS, as determined by intracellular flow cytometry staining, indicating differentiation of CiSCs in the presence of FBS. **c** Confocal immunofluorescence images for Desmin (green) and α-SMA (red) of control BM-MSCs and MCF7, Hela, and HepG2 CiSCs. Nuclei stained with DAPI (blue). Scale bars = 60 μM. **d** Flow cytometry overlay histogram analysis of CD34, CD19, CD45, and Oct-4 in BM-MSCs and MCF7, Hela, and HepG2 CiSCs following exposure to 10% FBS for 7 days, showing an increase in cell surface expression levels of CD34, CD19, and CD45 but a decrease in intracellular expression of Oct-4. For comparison, isotype control (black) was used to define the positive and negative population for each marker. **e** Schematic representation of the proposed model for MSC reprogramming to a cancer stem-like cell in the tumor microenvironment. Depiction of the proposed model in which MSCs are recruited to the tumor microenvironment where microenvironmental stimulus such as TGF-β can induce recruited MSCs to undergo reprogramming to a cancer stem cell phenotype enabling tumor progression and metastasis and modulating drug sensitivity. Taken together, these results suggest a critical role for the tumor microenvironment in determining the fate of the MSCs recruited to the tumor microenvironment. BM-MSC bone marrow mesenchymal stem cell, CSC cancer stem cell, CiSC cancer-induced stem cell, TGF-β transforming growth factor beta

The appearance of SP cells expressing ESC markers in CiSCs, together with the diverse morphology seen in cells derived from these spheres, led us to question the evidence for their differentiation into all three embryonic layers. CiSC spheres were transferred into adherent plates and cultured in standard DMEM supplemented with 10% FBS for real-time qPCR analysis of mRNA expression. This serum-containing culture medium has been shown previously to result in loss of tumor-initiating capacity of the CSCs and induces differentiation [73, 74]. mRNA for markers of all three embryonic layers was detected in spheres and CiSCs (Fig. 7a). These markers included important developmental transcription factors such as βIII-tubulin (a marker of ectoderm), α-fetoprotein (AFP, endoderm), glial fibrillary acidic protein (GFAP, ectoderm), forkhead box A2 (FOXA2, endoderm), paired box 6 (PAX6, ectoderm), Msh homeobox 1 (MSX1, mesoderm), and SRY-box containing gene 17 (SOX17, endoderm) (Fig. 7a). In contrast, expression of OCT3/4 was markedly decreased in the cells cultured in FBS-supplemented media as determined by intracellular flow cytometry staining (Fig. 7b).

Following their culture in FBS supplemented media, CiSCs were shown to be highly positive for markers of terminally differentiated cells such as α-SMA and Desmin, as determined by immunofluorescence confocal imaging (Fig. 7c). Flow cytometry of CiSCs showed that approximately 29% of the population expressed the hematopoietic stem cell marker CD34, 16% expressed the B-cell marker CD19, and approximately 30% expressed the hematopoietic marker CD45 (Fig. 7d and Additional file 1: Figure S1C, D). Spheroid CiSCs that were supplemented with FBS in adherent plates for up to 21 days were initially adherent. After 1 week in culture, they differentiated into large, polygonal epithelial-like cells, a phenotype similar to that of the adherent cancer cell line cultures (Additional file 1: Figure S1A). Importantly, when CiSC spheroids were cultured in the presence of FBS in Matrigel, which represents a reconstituted 3D culture system, the generated colonies differentiated and formed complex secondary structures (Additional file 1: Figure S1B). The formation of these complex secondary structures on Matrigel may indicate their differentiation capacities as described previously [75]. These data demonstrated that CiSCs could differentiate into the three germ layers in vitro.

### Cytoskeleton organization and ultrastructural characterization of CiSCs

**Fig. 8:**
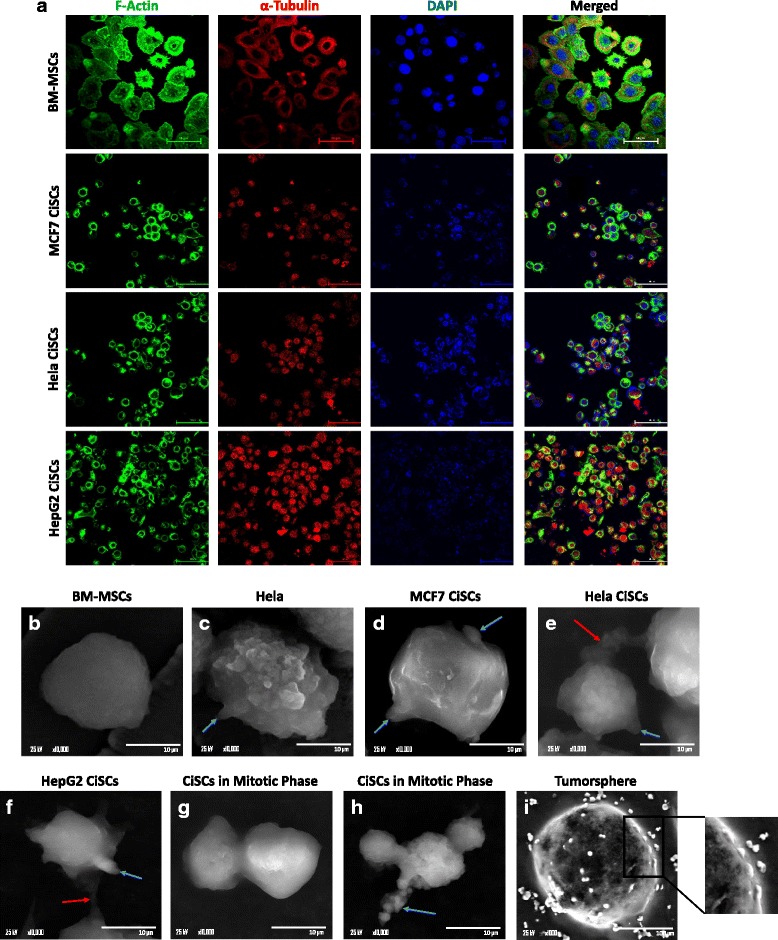
Cytoskeleton organization and surface ultrastructural characterization of CiSCs. **a** Confocal immunofluorescence staining of the actin cytoskeleton using phalloidin (green) and α-tubulin (red) showing localization of actin around the cell periphery while the α-tubulin network was distributed throughout the cell. Nuclei stained with DAPI (blue). Scale bars = 60 μM. **b**–**i** SEM revealed parental BM-MSCs to have a smooth and uniform surface (**b**) while (**c**) Hela cancer cells and (**d**) MCF7, (**e**) Hela, and (**f**) HepG2 CiSCs had an irregular surface and many microvilli and protrusions in the form of tumor-like buds (blue arrows). Adjacent cells interconnected by active pseudopodia (red arrows). **g**, h Mitotic cell division phase of CiSCs showing apophysis. (**i**) SEM of a tumorsphere and magnification showing tumor-like buds on the surface of the tumorsphere. BM-MSC bone marrow mesenchymal stem cell, CiSC cancer-induced stem cell

The cytoskeleton is known to have many roles in motility, invasion, polarity, survival, and growth of normal cells. Recent reports, however, demonstrated that the cytoskeleton is usually subverted in cancer cells to contribute to cancer cell growth, stiffness, movement, and invasiveness [76]. Recent reports show that cancerous cells exhibit an increasing deformability pattern and biomechanical homogeneity as they transition into more aggressive phenotypes [77]. This transformation is associated with changes in the actin cytoskeleton, with little to no effect on the microtubule network [77], suggesting that cell stiffness is inversely related to tumorigenesis and metastatic potential [78]. We thus characterized the actin and microtubule network in the CiSCs. In accordance with previous studies [79], our data show that the cytoskeleton of CiSCs displayed more deformability compared to their parental BM-MSCs. This is shown by localization of the actin filaments around the cell periphery, while no change in the tubulin network was observed (Fig. 8a). The localization of actin filaments around the cell periphery has been shown recently to act as a cage protecting the cellular contents from environmental insults and damage when migrating through tiny spaces [80].

Many reports have shown that the surface ultrastructure of cancer cells is unique and important for cancer development and can predict cell–cell and cell–matrix interactions, adhesion, and migration abilities [81, 82]. Accordingly, we analyzed the surface and cellular ultrastructure of CiSCs and their parental BM-MSCs as well as control Hela cells. SEM showed the parental BM-MSCs to have a smooth and uniform surface with no protrusions (Fig. 8b). However, the CiSCs showed many protrusions in the form of micro buds on the cell surface, with distinct microvilli (Fig. 8d–f), similar to the surface of Hela cancer cells (Fig. 8c). In the mitotic division phase of CiSCs, the cells were apophysis shaped (Fig. 8g, h) and adjacent cells were interconnected by active pseudopodia. SEM analysis of tumorspheres derived from CiSCs revealed tumor-like buds on their surface (Fig. 8i). These tumor-like buds have been recently shown to represent a population of migrating CSCs that have undergone EMT [83] and we are currently in the process of determining the development of these tumor-like buds and their role in tumor progression.

### Functional analysis of CiSCs in vivo in nude mice

To determine whether CiSCs generated from BM-MSCs may have the tumor-forming capability of cancer stem cells, CiSCs cultured for 7 days in suspension were injected under the kidney capsule of nude mice. CiSCs failed to form tumors in vivo after 2 months (Additional file 1: Figure S2). However, several reports showed that tumor formation in vivo is not always the result of injecting CSCs. For example, the work by Quintana et al. [84] shows that after injection of single, unselected melanoma cells, 27% of melanoma cells initiated a tumor, suggesting that the frequency of rare cancer-initiating cells is so far significantly underestimated. This is also supported by the study from Kelly et al. [85], who showed that regardless of the number of lymphoma cells injected, all animals developed a tumor even when transplanting only a single neoplastic cell, suggesting that tumor growth need not be driven by CSCs. Other work showed increased efficiency of transplantation and tumor formation when the cell suspension was mixed with Matrigel (a basement membrane-like substance that contains many growth factors) prior to implantation [86]. These data support the notion that the interaction between tumor cells and their microenvironment is critical for tumor formation and cellular engraftment. Recently, scientists have argued that the xenotransplantation assays select for cells more fit to grow in a foreign and hostile environment [84, 87]. Some cells with tumor initiation activity in humans may thus not display growth as xenografts. Additionally, the fact that most CSC surface markers are, in one way or another, linked to cellular attachment supports the view that CSC representation is dependent on the tumor microenvironment [88]. Because of these findings, the term “tumor-initiating cells” has been adopted instead of CSCs to refer to the tumor-initiating and propagating cells [89]. Along with recent studies showing that the preferential engraftment of CSCs was not observed in some mouse models [85, 90], our in-vivo results suggest the critical role of the tumor microenvironment in sustaining the tumorigenic potential of CiSCs in vivo. The long-term maintenance of the tumorigenic potential of CiSCs may thus depend on continuous exposure to cancer-inducing signals produced by cancer cells, at least in this experimental model. CiSC cell cycle analysis showed them to be quiescent and slow-cycling, and thus they may require more time for tumor development following xenotransplantation. Further experiments in our laboratory are ongoing to elucidate the tumorigenic potential of the CiSCs in vivo.

### Coculture of BM-MSCs with noncancerous cells does not generate CiSCs

As already discussed, soluble factors produced by cancer cells can induce morphological and genetic changes in BM-MSCs. To investigate whether soluble factors produced by normal, noncancerous cells can trigger a similar effect, BM-MSCs were cocultured with human dermal fibroblasts (HDF) as a model of noncancerous cells. After 5 days in this culture, no changes were observed in the morphology of the BM-MSCs (Additional file 1: Figure S3A). Moreover, no significant changes in mRNA expression of cancerous markers (Additional file 1: Figure S3B) or pluripotency markers (Additional file 1: Figure S3C) were found in the BM-MSCs after coculture with HDF. These results indicate that cancerous cells specifically produce factors that can trigger a CSC signature in BM-MSCs.

## Discussion

The relationship between host and tumor has recently been shown to be dynamic, whereby the environment of the host affects the behavior of the tumor, and the tumor influences the host. Several recent studies now suggest that CSCs may arise from either stem cells or progenitors [14, 15, 91, 92], or may be generated by dedifferentiation of somatic cells that acquire CSC-like properties under certain conditions [93–98].

Recent reports indicate that CSCs may originate from stem cells that have acquired malignant mutations [99–102]. However, other studies suggest that CSCs are in a state of flux and that microenvironmental stimulations can enrich the CSC population [103–105]. Herein, we provide several lines of evidence demonstrating that MCF7, Hela, and HepG2 cancer cells secrete soluble factors that induce phenotypic and genotypic changes in BM-MSCs via a paracrine effect. Incubation of BM-MSCs with cancer cells induced the following changes in the somatic MSCs: generated proliferating sphere-like cells in suspension; upregulated the expression of pluripotency markers Oct4, Sox2, and Nanog, and maintained this expression profile in culture; generated cells with high nonadherent colony-forming ability and increased sphere formation capability; increased expression of CSCs and cancer-related genes; produced a CD44^+^CD24^low^ CSC phenotype; generated cells with SP properties; increased the expression of ALDH1A1 and β-catenin; generated slow-cycling chemoresistant cells with low DNA damage response; generated cells that could differentiate into all three lineages and formed complex secondary structures when cultured on Matrigel; generated cells with a more pronounced mesenchymal phenotype than their parental BM-MSCs; and, with microenvironmental stimulation with TGF-β, further stimulated their cancerous properties and increased their sphere formation and invasion properties. Because some of these characteristics are related to acquiring CSC features, our study may provide evidence for the direct effect of the cancer microenvironment on generating the CSC phenotype.

MSCs have an important microenvironmental role in modulating tumor progression and drug sensitivity [9–11]. Recent reports demonstrated that MSCs are recruited in large numbers to the stroma of developing tumors [5–8]. Other studies showed that administration of BM-MSCs with breast cancer cells increased the tumor size and enhanced metastatic capacity by about 10-fold [106, 107], suggesting that differentiated tumor cells may fail to create the right environment and need an appropriate microenvironment to display tumor-initiating capacity [108]. However, the fate of the MSCs in the tumor microenvironment and the mechanism of supporting tumor growth remain unclear. It is therefore imperative to understand the bidirectional communication between tumor cells and MSCs within the tumor stroma. Cancer cells alone cannot drive tumor growth or progression; instead, assemblages of normal tissue and bone marrow-derived stromal cells are recruited to constitute tumorigenic microenvironments [1]. Most of the recruited cells in the tumor microenvironment are then coopted by the tumor to acquire and transit into tumor-associated stromal cells in order to support tumor progression and growth [109]. Recent reports provide convincing evidence that tumor-associated macrophages [110], cancer-associated fibroblasts [111, 112], and myeloid-derived suppressor cells [113, 114] are tumor cells derived from normal cells, recruited to the site of malignancy. Our data further support these findings, and suggest that MSCs recruited to the tumor microenvironment are exposed to cancer cell-secreted factors and may transition into more stem cell-like cells and acquire some CSC properties, and accordingly may contribute to the origin of CSCs.

Emerging evidence suggests that the MSC source and status might contribute to cancer cell behavior. For example, the work of Castellone et al. [115] has shown recently that direct MSC–cancer cell coculture resulted in an interesting physical interaction via membrane protrusions between the two cell populations, where cancer cells can absorb the MSCs leading to a more aggressive metastatic cell. Another report by Bartosh et al. [116] showed that in 3D direct coculture MSCs surrounded breast cancer cells and promoted the formation of cancer spheroids, leading to phagocytosis of MSCs by breast cancer cells. This engulfing promoted dormancy and the activation of prosurvival factors in the tumor, which is indeed a characteristic of CSCs. In this study, we report a similar effect but through a paracrine effect in an indirect coculture, without cell–cell contact. In this culture condition, soluble factors produced by cancer cells generated spheroid-like cells with many properties of CSCs. Our findings that soluble factors can covert non-CSCs to cells with a CSC signature are in line with relevant studies on the contribution of the tumor microenvironment to converting normal cells to cells with CSC properties through secreted soluble factors. For instance, endothelial cell-conditioned media were shown to produce the CSC phenotype in colorectal cancer cells [117], and myofibroblast-secreted factors conferred the CSC phenotype on differentiated cancer cells [105]. Other studies demonstrated that hypoxia-inducible factors (HIFs) can induce a CSC phenotype [118].

## Conclusions

Our findings herein may have far-reaching consequences in cancer therapy. Current strategies aim at attacking the tumor at its root, by developing CSC-selective therapies [119]. Our data also suggest that investigating the role of cancer-secreted factors, and not only cancer cells in promoting the disease and in therapy, should be of high priority. If cancer cell-specific factors can confer a more stem cell-like state with CSC characteristics on the recruited MSCs in the tumor stroma, then the approach of targeting only CSCs may fail to eradicate the cancer. Ongoing regeneration of new CSCs from the recruited MSCs, stimulated by the infectious properties of cancer cells, will continue the vicious cycle (Fig. 7e). The model we propose here indicates that therapeutic targeting should be directed to the microenvironmental factors, produced by cancer cells through their interaction with recruited MSCs, which contribute to the regeneration of CSCs. In addition to targeting CSCs, therapeutic approaches to cancer should focus on the cancer microenvironment.

Our findings also hold implications for development of anticancer therapeutics. We show that large-scale generation of chemoresistant cancer stem-like cells can be produced by coculturing BM-MSCs with cancerous cells without any genetic manipulations. The generated chemoresistant stem-like cells may be used for high-throughput screening for candidate therapeutic agents that specifically target CSCs. These cells can also be useful for studying the disease mechanism, biology, and toxicology. Further research in our laboratory is ongoing to determine specific factors and mechanisms responsible for the observed development of cancer stem-like cells from MSCs, with the promise of developing novel targets for cancer therapies aimed at targeting CSCs.

## Additional file

Additional file 1: Figure S1. showing in-vitro differentiation of CiSCs, **Figure S2.** showing xenotransplantation of CiSCs under the kidney capsule of nude mice, **Figure S3.** showing effect of coculturing BM-MSCs with HDF, and **Table S1.** presenting sequences of primers used. (PDF 1426 kb)

## Abbreviations

ALDH1A1: Aldehyde dehydrogenase 1 family member A1
bFGF: Basic fibroblast growth factor
BM-MSC: Bone marrow mesenchymal stem cell
CiSC: Cancer-induced stem cell
CSC: Cancer stem cell
EDTA: Ethylenediaminetetraacetic
EGF: Epidermal growth factor
EMT: Epithelial–mesenchymal transition
ESC: Embryonic stem cell
HDF: Human dermal fibroblasts
hTERT: Telomerase reverse transcriptase
MSC: Mesenchymal stem cell
PI: Propidium iodide
SP: Side population
TGF-β: Transforming growth factor beta
