# Supplementary material for: Cancer cell-soluble factors reprogram mesenchymal stromal cells to slow cycling, chemoresistant cells with a more stem-like state
Source: Stem Cell Res Ther. 2017 Nov 7;8:254. doi: 10.1186/s13287-017-0709-9 (PMC5688803; doi:10.1186/s13287-017-0709-9)
Supplement: Additional file 1: Figure S1. — showing in-vitro differentiation of CiSCs, Figure S2. showing xenotransplantation of CiSCs under the kidney capsule of nude mice, Figure S3. showing effect of coculturing BM-MSCs with HDF, and Table S1. presenting sequences of primers used. (PDF 1426 kb) [file 13287_2017_709_MOESM1_ESM.pdf]

Supplementary Figure 1

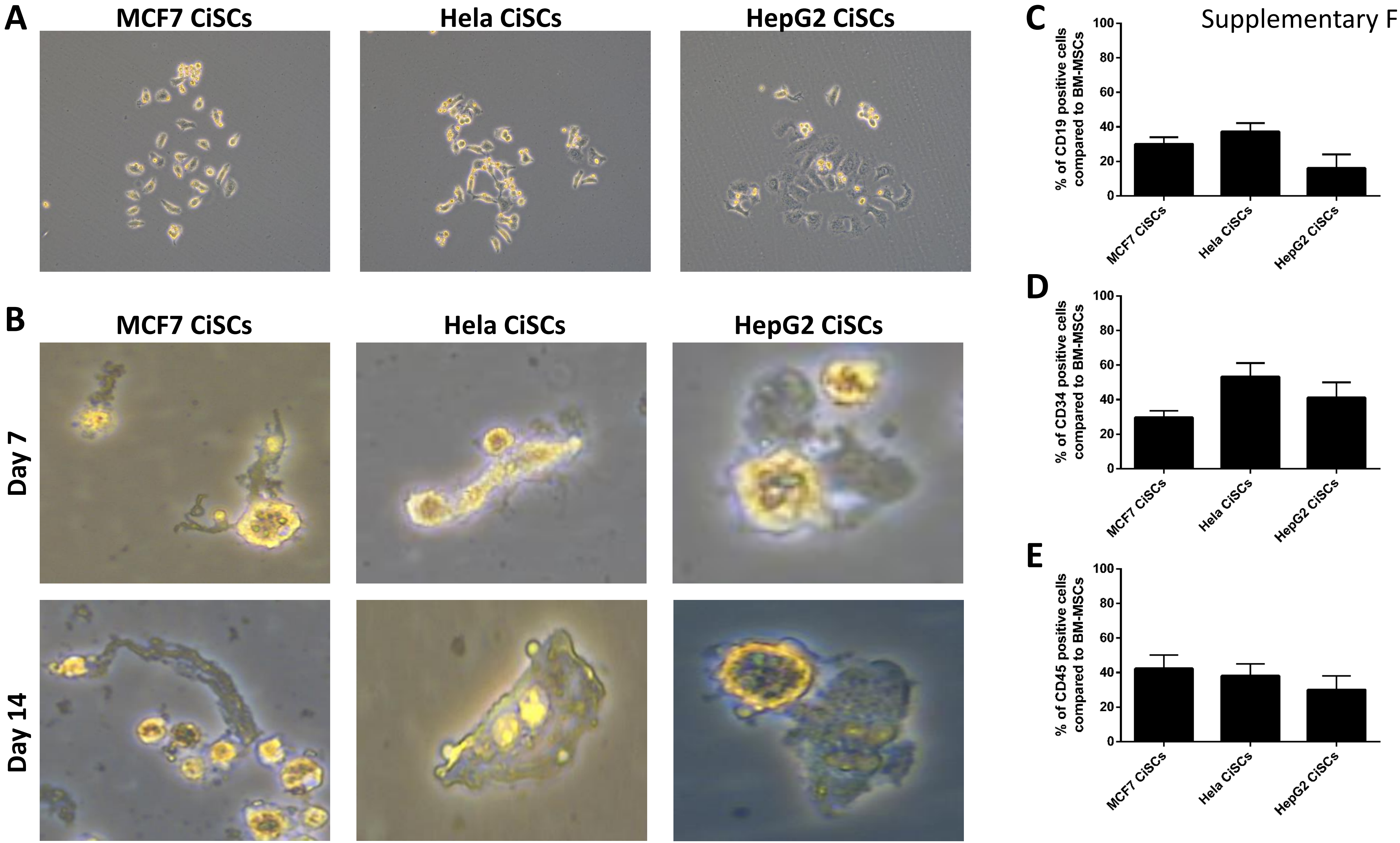

Supplementary Figure 2

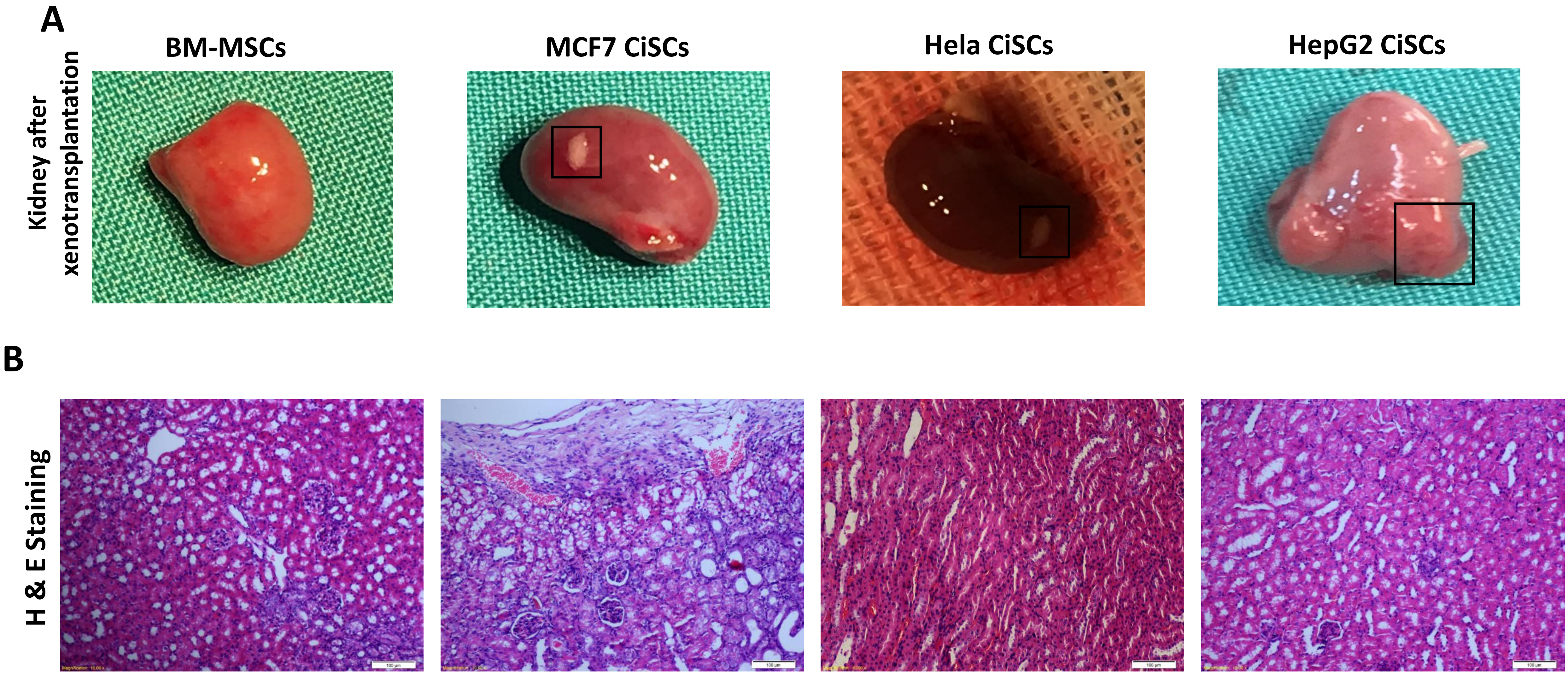

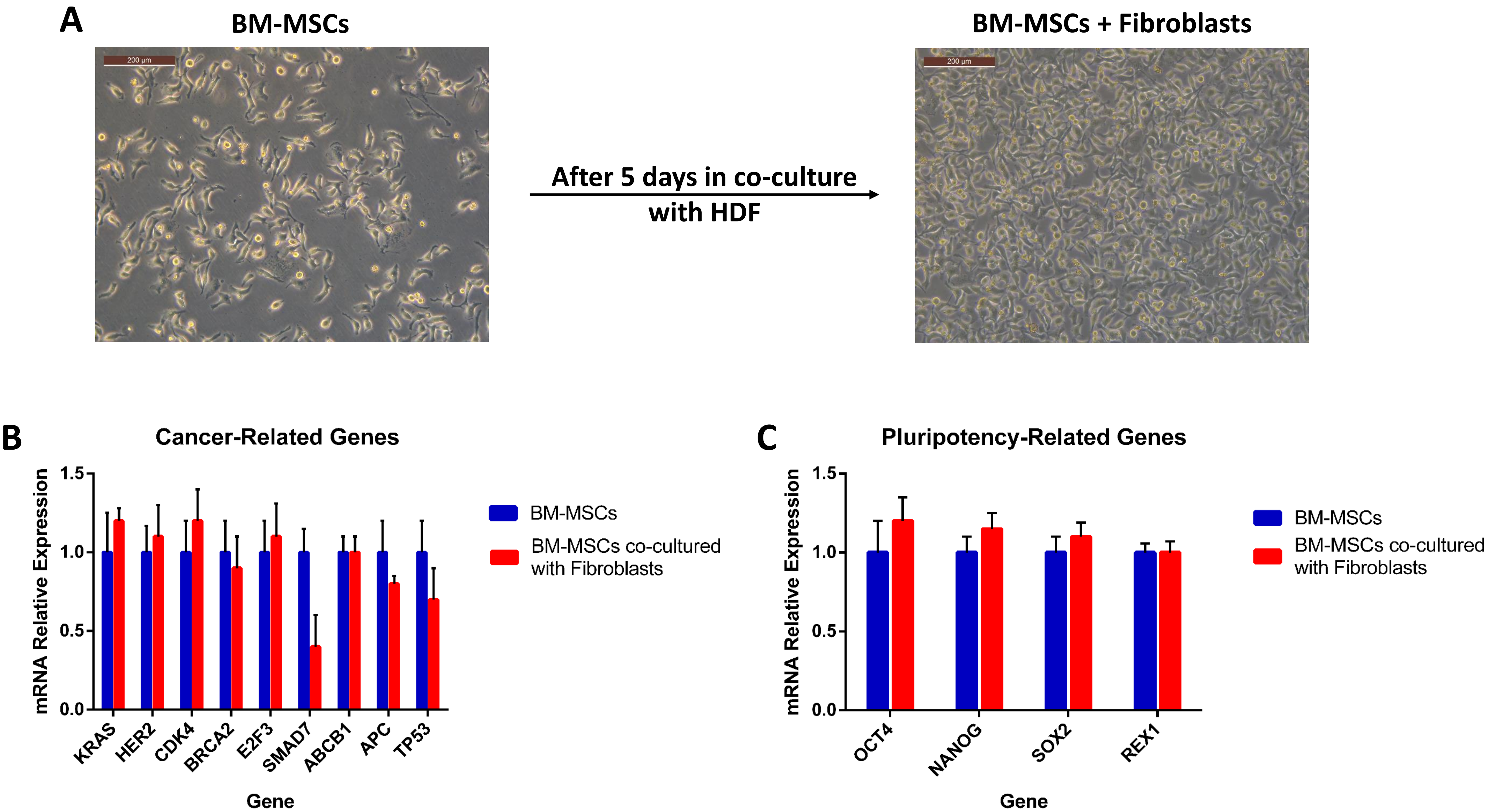
A
BM-MSCs
200 µm
After 5 days in co-culture
with HDF
BM-MSCs + Fibroblasts
200 µm
B
Cancer-Related Genes
mRNA Relative Expression
1.5
1.0
0.5
0.0
KRAS
HER2
CDK4
BRCA2
E2F3
SMAD7
ABCB1
APC
TP53
Gene
BM-MSCs
BM-MSCs co-cultured
with Fibroblasts
C
Pluripotency-Related Genes
mRNA Relative Expression
1.5
1.0
0.5
0.0
OCT4
NANOG
SOX2
REX1
Gene
BM-MSCs
BM-MSCs co-cultured
with Fibroblasts

Supplementary Figure 1: In vitro differentiation of CiSCs.

(A) Representative morphological analysis of CiSCs differentiation following exposure to 10% FBS for up to 3 weeks in adherent-mediated flasks.

(B) Phase-contrast images of CiSCs differentiation in matrigel (7-14 days) showing the formation of complex secondary structures.

(C-E) Quantification of the percentage of (C) CD19 (D) CD34 and (E) CD45 positive cells compared to the parental BM-MSCs by flow cytometry staining. Proportions of positive cells were measured by subtracting the control parental BM-MSCs staining from test histograms using super-enhanced Dmax (SED) normalized subtraction using FlowJo v. 10.2 software. Data are represented as mean ± SD.

Supplementary Figure 2: Xenotransplantation of CiSCs under the kidney capsule of nude mice.

(A) Representative images of kidneys of nude mice after 2 months following xenotransplantation of CiSCs.

(B) H&E staining of paraffin-embedded sections of kidneys of nude mice after 2 months following xenotransplantation of CiSCs.

Supplementary Figure 3: Effect of co-culturing BM-MSCs with Human Dermal Fibroblasts.

(A) Phase-contrast images of BM-MSCs at day 0 and after 5 days following co-culture

(B-C) Real-time qRT-PCR analysis of (B) cancer-related genes and (C) pluripotency-related genes in BM-MSCs following 5 days of co-culture with HDF. β-actin mRNA was used to normalize the variability in template loading. The data are reported as mean ± SD.

Supplementary Table S1: Sequence of Primers used

Gene Name	Primer Sequence
Human β-actin Forward	AGAGCTACGAGCTGCCTGAC
Human β-actin Reverse	AGCACTGTGTTGGCGTACAG
Human APC Forward	AGCCATGCCAACAAAGTCATCACG
Human APC Reverse	TTCCTTGCCACAGGTGGAGGTA
Human CDK4 Forward	TCGAAAGCCTCTCTTCTGTG
Human CDK4 Reverse	TACATCTCGAGGCCAGTCAT
Human TP53 Forward	GTTCCGAGAGCTGAATGAGG
Human TP53 Reverse	TTATGGCGGGAGGTAGACTG
Human E2F3 Forward	GAGACTGAAACACACAGTCC
Human E2F3 Reverse	CCTGAGTTGGTTGAAGCC
Human SMAD7 Forward	AGAAGGTGCGGAGCAAAAT
Human SMAD7 Reverse	GTGTGGCGGACTTGATGA
Human ABCB1 Forward	AAATTGGCTTGACAAGTTGTATATGG
Human ABCB1 Reverse	CACCAGCATCATGAGAGGAAGTC
Human BRCA2 Forward	CGAGCTTCTGAAACTAGGC
Human BRCA2 Reverse	CTTTGTTGCAGCGTGTCTTA
Human HER2 Forward	ATCTGCCTGACATCCACG
Human HER2 Reverse	GCAATCTGCATACACCAGTTC

Human KRAS Forward	TGTTCACAAAGGTTTTGTCTCC
Human KRAS Reverse	CCTTATAATAGTTTCCATTGCCTTG
Human ALDH1A1 Forward	CGCAAGACAGGCTTTTCAG
Human ALDH1A1 Reverse	TGTATAATAGTCGCCCCCTCTC
Human CD44 Forward	AGAAGGTGTGGGCAGAAGAA
Human CD44 Reverse	AAATGCACCATTTCCTGAGA
Human ABCG2 Forward	TTTCCAAGCGTTCATTCAAAAA
Human ABCG2 Reverse	TACGACTGTGACAATGATCTGAGC
Human PTEN Forward	CAAGATGATGTTTGAAACTATTCCAATG
Human PTEN Reverse	CCTTTAGCTGGCAGACCACAA
Human CD90 Forward	TCAGGAAATGGCTTTTCCCA
Human CD90 Reverse	TCCTCAATGAGATGCCATAAGCT
Human Nestin Forward	AGCGTTGGAACAGAGGTTGGA
Human Nestin Reverse	TGTTTCCTCCCACCCTGTGTC
Human CD24 Forward	TGCTCCTACCCACGCAGATT
Human CD24 Reverse	GGCCAACCCAGAGTTGGAA
Human EpCAM Forward	CGCAGCTCAGGAAGAATGTG
Human EpCAM Reverse	TGAAGTACACTGGCATTGACG
Human Oct4 Forward	TGTACTCCTCGGTCCCTTTC
Human Oct4 Reverse	TCCAGGTTTTCTTTCCCTAGC

Human Sox2 Forward	GCTAGTCTCCAAGCGACGAA
Human Sox2 Reverse	GCAAGAAGCCTCTCCTTGAA
Human Nanog Forward	CAGTCTGGACACTGGCTGAA
Human Nanog Reverse	CTCGCTGATTAGGCTCCAAC
Human Rex1 Forward	GCGTACGCAAATTAAAGTCCAGA
Human Rex1 Reverse	ATCCTAAACAGCTCGCAGAAT
Human hTERT Forward	CGGAAGAGTGTCTGGAGCAA
Human hTERT Reverse	GGATGAAGCGGAGTCTGGA
Human Bcl-2 Forward	GGATAACGGAGGCTGGGATG
Human Bcl-2 Reverse	TGACTTCACTTGTGGCCCAG
Human Bax Forward	CAAACTGGTGCTCAAGGCCC
Human Bax Reverse	GAGACAGGGACATCAGTCGC
Human PARP Forward	AGCGTGTTTCTAGGTCGTGG
Human PARP Reverse	CATCAAACATGGGCGACTGC
Human N-Cadherin Forward	GGTGGAGGAGAAGAAGACCAG
Human N-Cadherin Reverse	GGCATCAGGCTCCACAGT
Human SNAIL Forward	ACCACTATGCCGCGCTCTT
Human SNAIL Reverse	GGTCGTAGGGCTGCTGGAA
Human ZEB1 Forward	GATGATGAATGCGAGTCAGATGC
Human ZEB1 Reverse	CTGGTCCTCTTCAGGTGCC

Human Twist1 Forward	CGGGAGTCCGCAGTCTTA
Human Twist1 Reverse	GCTTGAGGGTCTGAATCTTG
Human Occludin Forward	CATTGCCATCTTTGCCTGTG
Human Occludin Reverse	AGCCATAACCATAGCCATAGC
Human Desmoplakin Forward	CAGTGGTGTCAGCGATGATGT
Human Desmoplakin Reverse	TGACGCTGGATATGGTGGAA
Human E-Cadherin Forward	GTCACTGACACCAACGATAATCCT
Human E-Cadherin Reverse	TTTCAGTGTGGTGATTACGACGTTA
Human FOXA2 Forward	CCGTTCTCCATCAACAACCT
Human FOXA2 Reverse	GGGGTAGTGCATCACCTGTT
Human AFP Forward	AGCAGCTTGTTAAATCAACATGCA
Human AFP Reverse	AAAATTAACTTTGGTAAACTTCTGACTCAGT
Human SOX17 Forward	CGCTTTCATGGTGTGGGCTAAGGACG
Human SOX17 Reverse	TAGTTGGGGTGGTCCTGCATGTGCTG
Human βIII-tubulin Forward	GCTCAGGGGCCTTTGGACATCTCTT
Human βIII-tubulin Reverse	TTTTCACACTCCTTCCGCACCACATC
Human GFAP Forward	AGAAGCTCCAGGATGAAACC
Human GFAP Reverse	AGCGACTCAATCTTCCTCTC
Human PAX6 Forward	TGGTATTCTCTCCCCCTCCT
Human PAX6 Reverse	TAAGGATGTTGAACGGGCAG

Human MSX1 Forward	CCTCTTTGCTCCCTGAGTTCA
Human MSX1 Reverse	GGGACTCTTCCAGCCACTTTTT
